# Occupational radiation exposure indicated by increased chromosomal damage in lymphocytes of orthopaedic surgeons in Japan

**DOI:** 10.1093/jrr/rraf085

**Published:** 2026-01-13

**Authors:** Donovan Anderson, Valerie Swee Ting Goh, Yohei Fujishima, Ryo Nakayama, Naoki Echigoya, Yasuyuki Ishibashi, Tomisato Miura

**Affiliations:** Department of Risk Analysis and Biodosimetry, Institute of Radiation Emergency Medicine, Hirosaki University, 66-1 Hon-cho, Hirosaki, Aomori 036-8564, Japan; Department of Radiobiology, Singapore Nuclear Research and Safety Initiative (SNRSI), National University of Singapore, 16 Prince George’s Park, Singapore, 118415, Singapore; Department of Risk Analysis and Biodosimetry, Institute of Radiation Emergency Medicine, Hirosaki University, 66-1 Hon-cho, Hirosaki, Aomori 036-8564, Japan; Department of Radiation Life Sciences School of Medicine, Fukushima Medical University, 1-Hikarigaoka, Fukushima-city, Fukushima 960-1295, Japan; Department of Orthopaedic Surgery, Tokiwakai Hospital, 2-1 Kameda, Sakaki, Fujisaki-machi, Aomori 038-1216, Japan; Department of Orthopaedic Surgery, Hirosaki University School of Medicine, 5 Zaifu-cho, Hirosaki, Aomori, 036-8564, Japan; Department of Risk Analysis and Biodosimetry, Institute of Radiation Emergency Medicine, Hirosaki University, 66-1 Hon-cho, Hirosaki, Aomori 036-8564, Japan

**Keywords:** chromosome aberration, cytogenetics, occupational exposure, fluoroscopy

## Abstract

This study aims to assess chromosome aberrations in peripheral blood lymphocytes of orthopaedic surgeons in Japan, specifically focusing on potential occupational dose overexposure and its correlation with adverse health reactions. The main objective is to investigate the extent of chromosomal damage and evaluate the accuracy of estimating radiation dose with cytogenetic biodosimetry where no physical dosimetry exists. This study involved 18 male orthopaedic surgeons, with occupational experience spanning 15 to 33 years. Chromosome aberrations were analyzed in 32 573 and 45 674 cells with dicentric chromosome and translocation assays, respectively. Statistical tests were used to retrospectively estimate whole-body doses with chromosome damage and compare observed aberration frequencies with work experience, while considering factors such as adverse health effects and skin cancer history. Materials and methods included information on study design, participant criteria and the procedures performed, using a retrospective approach. Participants had a mean age of 46 ± 6.6 years. Analysis revealed an increase in dicentric abnormalities compared to background levels, and translocations were observed above spontaneous levels in all surgeons but one. Surgeons reporting adverse health effects or skin cancer exhibited the highest chromosome aberrations frequencies. The estimated average whole-body doses were 75 ± 24 and 321 ± 103 mGy for dicentrics and translocations, respectively. Some Japanese orthopaedic surgeons demonstrated increased chromosome aberrations, especially in those reporting adverse health effects. Estimating radiation dose solely based on chromosomal damage is challenging, emphasizing the complexities of biological dosimetry for prior, partial and repeated exposures.

## INTRODUCTION

**Fig. 1:**
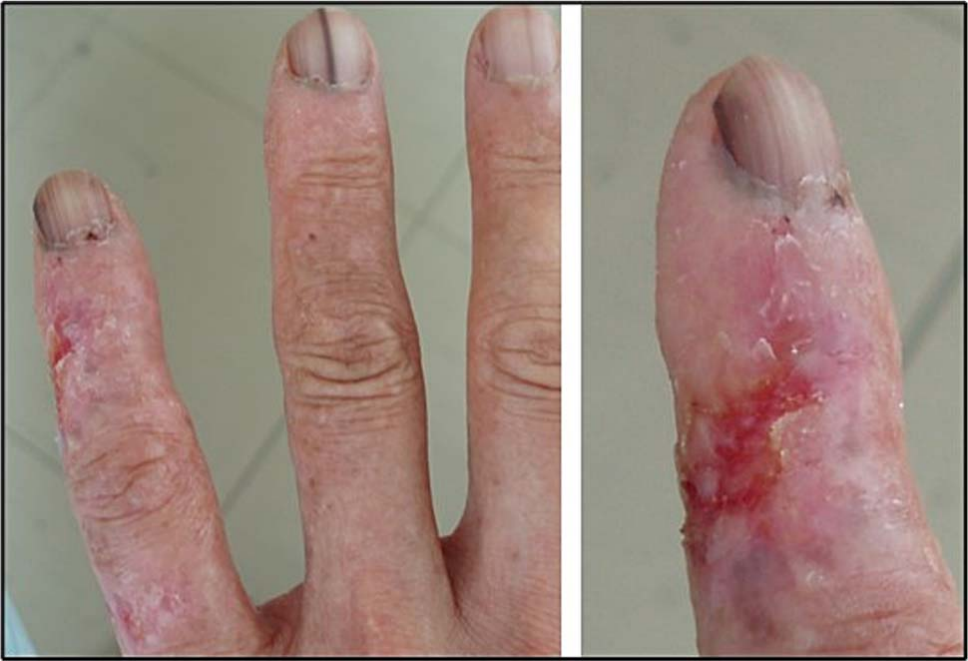
Photograph of the hand of an orthopaedic surgeon participating in this study (HKX001), showing skin damage, melanonychia of nails, and diagnosed squamous cell carcinoma, which is a sign of fractionated, localized radiation exposure. The image was taken by one of the co-authors with the surgeon’s consent, and no external copyright applies.

Orthopaedic surgeons are Category A radiation workers at risk of occupational exposure to ionizing radiation when performing interventional fluoroscopy [1]. Even though most orthopaedic surgeons report doses below international safety limits [2], severe tissue reactions can still occur from high levels of radiation due to malpractice, such as those shown in Fig. 1. Repeated exposure to radiation, both high and low, can increase the risk of late and other adverse effects including leukemia and solid cancer [3–5]. In particular, orthopaedic surgeons in Japan face significant radiation risks during procedures like balloon kyphoplasty and spine surgeries that require fluoroscopy [6–8]. A 2022 survey among spine surgeons in Japan performing ≥36 procedures yearly found that ~50% reported inconsistent use of some personal protective equipment (PPE), mainly gloves and thyroid shields, highlighting the need for improved awareness and implementation of radiation safety education programs [7, 8]. Medical professionals using fluoroscopy often lack formal training in radiation protection and may not have ready access to specialists like medical physicists [2, 9, 10]. However, assessing the occupational radiation dose for a surgeon is challenging due to various factors, including surgical techniques, imaging approaches, X-ray system configurations and improper dosimeter positioning [11].

Occupational radiation exposure can be monitored using physical dosimeters, such as thermoluminescent dosimeters, as well as cytogenetic biodosimetry methods like the dicentric chromosome assay [12, 13]. Biodosimetry is also useful when physical dosimetry is unavailable or inaccurate [14, 15]. Cytogenetic biodosimetry uses chromosome aberrations, like dicentric chromosomes and translocations, which increase with dose for whole-body exposures under uniform radiation fields and are indicative of DNA damage from ionizing radiation [16, 17]. Radiation-induced DNA double-strand breaks trigger complex repair mechanisms primarily involving non-homologous end joining and homologous recombination. Errors in these repair processes can result in structural chromosome aberrations. Dicentric chromosomes, formed when two chromosome fragments join to form a single chromosome with two centromeres, show high specificity for radiation exposure but are unstable and short-lived, limiting their utility for assessing historical exposures [18, 19]. In contrast, translocations, which involve the exchange of chromosomal segments between non-homologous chromosomes, are stable and persist for decades, making them more suitable for evaluating past or chronic exposures [20–22]. Confounding factors such as age and smoking are known to influence baseline translocations frequencies [23], while medical history can influence baseline chromosome aberrations [24].

In this study, both unstable and stable chromosome aberrations in peripheral blood lymphocytes of 18 male orthopaedic surgeons in Japan were studied. Adverse health effects such as hand dermatitis and necrosis possibly caused by high doses from direct exposure to the primary radiation beam, and skin cancer from cumulative low-dose exposures were also reported. Despite the surgeons’ reported adherence to occupational dose limits, cytogenetic biodosimetry provided some insight to their cumulative occupational radiation exposure and relative efficacy of each aberration for long-term dose monitoring.

## MATERIALS AND METHODS

### Blood collection and ethics

Human peripheral blood from 18 male orthopaedic surgeons (identified as HKX001 to HKX018, aged between 36 and 56 years) was collected in heparinized vacutainer tubes (Becton Dickinson) with informed consent. No exclusion criteria were applied for surgeon recruitment; all available orthopaedic surgeons who consented to participate were included regardless of experience or gender. This study was reviewed and received ethical approval by the Committee of Medical Ethics of the authors’ institution (Organizing No. 2016–011). All active surgeons were working in different hospitals in Japan and completed a questionnaire (Supplementary Fig. S1). Information about donors were recorded, including smoking habits, years of experience and any adverse health effects (Table 1). The surgeons had no known exposure to radiation within the past 12 months; however, all had performed fluoroscopy exams for patients and were exposed to X-ray radiation over a period of 11–33 years. None of the surgeons had access to or provided any records of their film badge personal dosimeter measurements or history. All surgeons reported that they had not been exposed to occupational radiation levels exceeding the Japanese occupational dose limits of 100 mSv per 5 years or 50 mSv in any single year. We did not inquire about their specific habits regarding the consistent use of PPE. However, each surgeon indicated that they wore a film badge personal dosimeter on their chest but did not use ring dosimeters to monitor partial doses to their hands or fingers. The Brinkman index (the number of cigarettes smoked per day multiplied by the number of years of smoking) was used to estimate the severity of smoking.

**Table 1:** Distribution of covariates among orthopaedic surgeons

Donor ID	Sex	Age	Radiation work (years)	Brinkman’s index	Reported adverse effect on skin and hands
HKX001	M	53	28	0	Squamous cell carcinoma
HKX002	M	50	24	310	Induration on fingernail
HKX003	M	44	19	220	Melanonychia on fingernail
HKX004	M	54	25	N/A^a^	No effects
HKX005	M	40	15	225	Melanonychia on fingernail
HKX006	M	45	18	100	Melanonychia on fingernail
HKX007	M	44	19	540	Bowen’s disease (early form of squamous cell carcinoma)
HKX008	M	49	24	0	Melanonychia on fingernail
HKX009	M	56	31	200	Melanonychia on fingernail
HKX010	M	40	13	100	Discoloration on fingernail
HKX011	M	38	11	0	Discoloration on fingernail
HKX012	M	51	25	0	Melanonychia on fingernail
HKX013	M	36	11	0	No effects
HKX014	M	41	15	N/A	No effects
HKX015	M	56	31	0	Induration on fingernail
HKX016	M	55	30	150	Squamous cell carcinoma
HKX017	M	43	18	N/A	Melanonychia on fingernail
HKX018	M	41	15	0	Induration on fingernail

^a^N/A indicates missing information from survey

### Lymphocyte isolation and cell culture

Whole blood was diluted in an equal volume of Roswell Park Memorial Institute (RPMI) washing media and then loaded onto an equal volume of Histopaque 1077 (Sigma-Aldrich) at room temperature, followed by centrifugation at 600 × *g* for 30 min at 20°C. The RPMI washing media was composed of RPMI 1640 supplemented with 2% heat inactivated fetal bovine serum (FBS, Sigma-Aldrich) and 60 μg/ml kanamycin sulphate (Thermo Fisher Scientific). The mononuclear cell fraction was transferred into RPMI washing media and washed once at 400 × *g* for 5 min at room temperature. Isolated lymphocytes were resuspended in RPMI complete media, then cell division was stimulated with 180 μg/ml phytohemagglutinin (PHA, HA-15 (Remel Europe)). To prevent the cells from progressing beyond the first metaphase, 0.05 μg/ml demecolcine (KaryoMAX™ Colcemid™ Solution in HBSS, Thermo Fisher Scientific) was added at the start of the culture. The RPMI complete media was composed of RPMI 1640 supplemented with 20% heat-inactivated FBS and 60 μg/ml kanamycin sulfate. Lymphocytes were cultured in a humidified 5% CO_2_ incubator at 37°C for 48 h.

### Chromosome preparation for Giemsa and fluorescence *in situ* hybridization

After culture, lymphocytes were harvested by centrifugation at 400 × *g* for 5 min at room temperature. The cell pellet was then resuspended in 75 mM KCl solution for 20 min at 37°C. Cells were washed in three changes of freshly prepared cold fixative (methanol: glacial acetic acid = 3:1 v/v). A final wash in fixative was done before dropping cells onto a pre-cleaned glass slide. Slides were then prepared for Giemsa staining or fluorescence *in situ* hybridization (FISH) labelling. Air-dried slides were stained in Giemsa solution (4%; Merck Millipore) for 10 min. For FISH labelling, slides were dried at 65°C for 1 h. FISH was done using whole chromosome painting of chromosomes 1, 2 and 4 by a customized XCP-Mix probe (Mix-#1R-#2G-#4RG; MetaSystems Probes), and nuclear counterstain with 4',6-diamidino-2-phenylindole (DAPI) (Vector Laboratories, Inc.), according to the manufacturer’s instructions [25, 26].

### Image capturing and chromosome aberration analysis

After Giemsa staining, metaphase images were captured using Axio Imager.Z2 fluorescence microscopes (Carl Zeiss AG) equipped with charge-coupled device cameras and Metafer4 software (version 3.11.8, MetaSystems). 

For FISH analysis, Metafer4 and the In Situ Imaging System FISH Imaging System software (Isis; version 5.5.10, MetaSystems) were used.

For Giemsa-stained slides, as close to 2000 metaphases for each donor were analyzed for chromosome aberrations (Supplementary Table S1). Only complete metaphases were scored (i.e. contained 45 or 46 centromeres, no chromosome overlap and distinguishable chromosome arms). Observable aberrations were classified as dicentrics, rings or excess fragments. Other chromosome- or chromatid-type aberrations, such as marker chromosomes, breaks and gaps, were recorded as ‘other’.

Dose estimations by dicentric frequencies were calculated using the formula Y_dicentric_ = C + αD + βD^2^, where Y_dicentric_ is the observed yield of dicentrics, D is absorbed dose in water and C, α, and β are background yield, linear and quadratic coefficients of the dose-response curve. The values 0.00153 (±0.00278), 0.0538 (±0.0155) and 0.0198 (±0.00435) were used for C, α and β, respectively. This dose-response curve was previously established using whole blood irradiated with X-rays, followed by lymphocyte culture and harvest [19].

For FISH-labelled slides, a minimum of 1000 cell equivalents (CE, ~3000 metaphases) were analysed for translocations, wherever possible (Supplementary Table S2). No metaphases were available for translocation scoring in one surgeon (HKX003) due to an insufficient volume of blood to perform both the dicentric and translocation assays. Only metaphases with all three pairs of differentially painted chromosomes were scored. Translocations were scored on Isis, according to the scoring criteria previously described [27]. In brief, one-way and two-way translocations were counted, and complex translocations were converted to equivalent number of simple translocations based on the number of colour junctions. The number of metaphases scored was converted to CE, using the formula based on three colours (chromosome 1: red; chromosome 2: green; chromosome 4: yellow) [28] and sex-specific individual chromosome lengths [29]. To compare translocations across donors, we obtained an age-corrected translocation frequency as described by Sigurdson *et al*. [23].

Dose estimations by translocation frequencies were calculated using the formula Y_translocation_ = C + αD + βD^2^, where Y is the age-corrected yield of translocations per CE, D is absorbed dose in water, and C, α and β are background yield, linear and quadratic coefficients of the dose-response curve. The values 0.0005 (±0.0001), 0.0178 (±0.0037) and 0.0901(±0.0054) were used for C, α and β, respectively [25, 27].

### Statistical analysis

Statistical analysis was performed using Minitab Statistical Software (ver. 21.4, Minitab). Significant differences between groups were determined using the Mann–Whitney *U*-test. *P*-value of < 0.05 was considered statistically significant. Correlation analysis was carried out by calculating the Pearson’s product-moment correlation coefficient. Multiple regression analysis was also conducted for both chromosome aberrations and factors such as age, smoking, and work experience.

## RESULTS

The study included a total of 18 male surgeons who were actively involved in performing fluoroscopy procedures at hospitals in Japan. The subjects were aged 46 ± 6.6 years (mean and standard deviation), ranging from 36 to 56 years. These surgeons had extensive work experience in the field, with an average of 21 ± 6.9 years, ranging from 11 to 33 years. These surgeons were selected as representative participants to assess the potential impact of occupational radiation exposure using chromosomal aberrations in peripheral blood lymphocytes, and to evaluate their accumulated exposure over their years of work. 15 surgeons reported adverse health effects (Table 1).

**Fig. 2:**
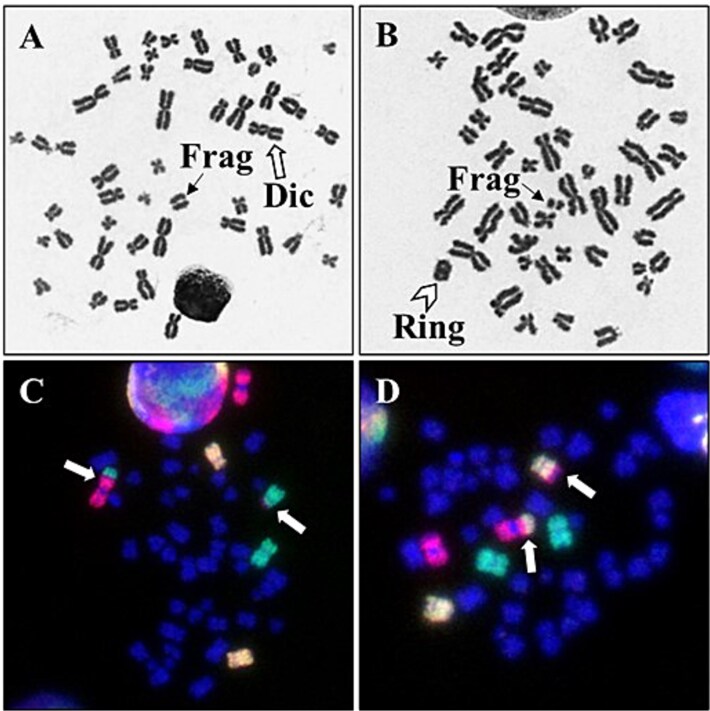
(A, B) show unstable chromosome aberrations of dicentric chromosomes, rings and their associated fragments observed in peripheral blood lymphocytes of orthopaedic surgeons. (C, D) show reciprocal translocations (arrow) observed by fluorescence in situ hybridization (FISH) labelling.

**Fig. 3:**
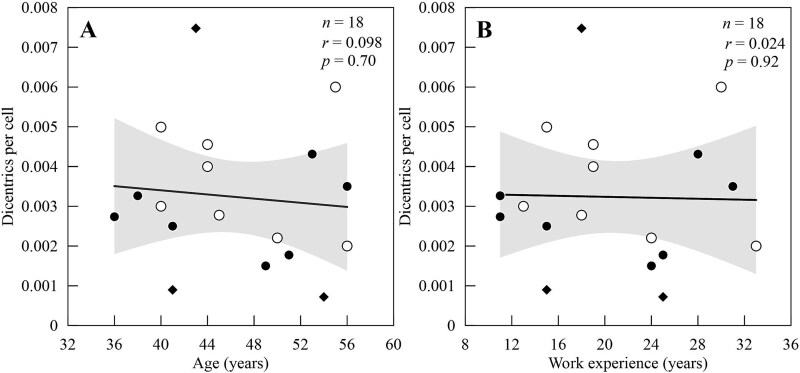
(A) Dicentric frequency per cell by age (years) and (B) by work experience (years) of male orthopaedic surgeons. In both graphs, closed black circles indicate non-smokers, open circles indicate smokers and the black diamond had no smoking information. The 95% confidence interval of all data is shown by the shaded grey area.

In the 18 individuals studied, a total of 32 573 metaphases were analysed using Giemsa-staining and 45 674 metaphases (18 031 CE) using FISH-labelling. Chromosome aberrations of dicentrics and translocations were observed in peripheral blood lymphocytes (Fig. 2). The average dicentric frequency per cell was 0.003 ± 0.002 (range, 0.001 to 0.007), while the average frequency of observed translocations per CE was 0.019 ± 0.009 (range, 0.008 to 0.036). In the case of dicentric chromosomes, all surgeons except two (HKX014 and HKX004) had dicentric frequencies higher than the general population’s background level of 0.001 dicentrics per cell [18, 25]. No significant differences in dicentric frequency and no correlation was observed with increasing age and work experience (*r* = 0.098, 0.024, respectively) (Fig. 3).

The estimated whole-body dose across all surgeons, calculated retrospectively based on cytogenetic markers, was 33 ± 30 mGy. Estimated individual doses ranged from 0 to 106 mGy (Supplementary Table S1). However, only 4 surgeons showed dicentric yield above decision threshold, and their estimated whole-body dose was 75 ± 24 mGy. Additionally, dicentric distributions followed a Poisson distribution and no over-dispersion was seen as *u* values were within −1.96 to 1.96 (detailed results not shown). The dicentric frequency was also weakly correlated to smoking tendencies (*r* = 0.29, Supplementary Fig. S2), though the relationship was not statistically significant (*P*-value = 0.30, Supplementary Fig. S2). Multiple regression analysis also showed that age, smoking and work experience did not influence dicentric frequency (Supplementary Table S3).

**Fig. 4:**
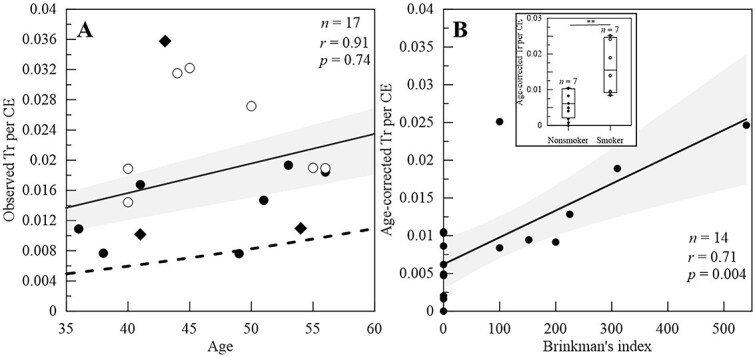
(A) Observed translocation frequency per cell equivalent by age. The systematically reviewed background translocation yields in normal, healthy humans by age, is provided by the dashed line. Closed black circles indicate non-smokers, open circles indicate smokers and the black diamond had no smoking information. (B) Age-corrected translocation frequency per cell equivalent by smoking habit, i.e., Brinkman’s index. Inset graph shows boxplot of age-corrected translocation per CE in non-smokers and smokers. In the boxplot, the center line represents the mean. In both graphs, the 95% confidence interval of all data is shown by the shaded grey area.

All surgeons analyzed for FISH, with the exception of one, demonstrated observed translocations per CE exceeding the spontaneous levels corresponding to their respective ages (Fig. 4A) [30]. Additionally, age-corrected chromosome translocations in peripheral blood lymphocytes significantly increased with the Brinkman’s index (i.e., cigarettes smoked per day) (Fig. 4B, *r* = 0.73, *P*-value < 0.001). Smokers had significantly higher translocation frequency per CE compared to non-smokers (Fig. 4B inset, *P*-value < 0.01). Similar to dicentric frequency, no correlation was observed between age-corrected translocations per CE and age, and work experience (*r* = 0.05, 0.09, Supplementary Fig. S3). Moreover, multiple regression analysis also showed that only smoking significantly influenced Tr frequency (*P*-value <0.01, Supplementary Table S4).

The estimated average whole-body dose across 17 surgeons based on yield of translocations was 231 ± 135 mGy (range, 0 to 473 mGy; Supplementary Table S2). Eight surgeons had observed translocations less than the decision threshold. If those surgeons are removed, the estimated average whole-body dose was 321 ± 104 mGy.

**Fig. 5:**
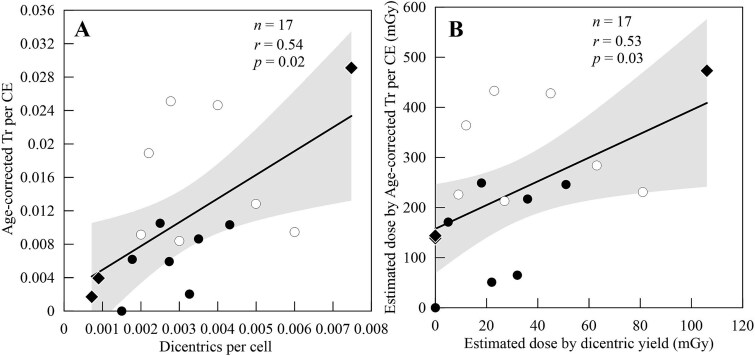
(A) Dicentric frequencies compared to age-corrected translocation frequencies in peripheral blood lymphocytes of orthopaedic surgeons. (B) Estimated doses by dicentric yield compared to estimated doses from age-corrected translocations per CE. In both graphs, closed black circles indicate non-smokers, open circles indicate smokers and the black diamond had no smoking information. In both graphs, the 95% confidence interval of all data is shown by the shaded grey area.

For dicentric and translocation frequencies, it is important to consider the time perspective for dose accumulation, as it remains unclear how much of the observed results can be attributed to direct exposure to the hands versus scattered radiation to the rest of the body. Furthermore, the frequency of translocations observed in surgeons was significantly higher than dicentrics, with up to nine times more translocations detected. We observed a significant positive linear relationship between the frequencies of dicentric chromosomes and age-corrected translocations, indicating a strong correlation between these two biomarkers (*r* = 0.54, Fig. 5A). Estimated doses by age-corrected translocation frequency was also higher for every surgeon when compared to those estimated by dicentric frequency (Fig. 5B).

The frequency of dicentrics and age-corrected translocations in the peripheral blood of surgeons who reported adverse health effects tended to be higher compared to those who did not report such effects (Table 2). The observed differences were found to be statistically significant (*P-value* < 0.05) for both biomarkers between the group with adverse effects and those with none reported. Surgeons diagnosed with squamous cell carcinoma (SCC) had the greatest average dicentric frequency among reported adverse effect groups.

**Table 2:** Frequencies of chromosome aberrations in surgeons compared to reported adverse effects

Adverse effect reported	Number of surgeons	Chromosome aberration frequencies (aberrations per 100 cells or 100 CE)				
		Dicentrics	Rings	Fragments	Translocations	
					Observed	Age-corrected
All surgeons with adverse effects	15	0.37 ± 0.2	0.06 ± 0.06	1.0 ± 0.4	2.1 ± 0.8	1.3 ± 0.8
Fingernail discoloration	3	0.31 ± 0.2	0.03 ± 0.03	1.1 ± 0.4	1.2 ± 0.26	0.6 ± 0.05
Melanonychia on fingernail	7	0.37 ± 0.1	0.05 ± 0.08	0.90 ± 0.3	2.1 ± 0.9^a^	1.4 ± 0.9 ^a^
Induration on finger, verruca	2	0.28 ± 0.4	0.09 ± 0.03	1.0 ± 0.8	2.1 ± 0.6	1.3 ± 0.72
Squamous cell carcinoma	2	0.51 ± 0.6	0.07 ± 0.05	1.1 ± 1.3	1.9 ± 0.4	1.0 ± 0.06
Bowen’s disease	1	0.40	0.10	1.0	3.2	2.5
Surgeons with no adverse effects	3	0.16 ± 0.1	0.05 ± 0.03	0.57 ± 0.1	1.1 ± 1.25	0.4 ± 1.3
All surgeons	18	0.32 ± 0.2	0.06 ± 0.6	0.94 ± 4.2	1.9 ± 0.9 ^a^	1.19 ± 0.9^a^

^a^One surgeon was excluded as no metaphase cells was obtained for translocation scoring.

## DISCUSSION

Numerous studies have examined cytogenetic markers for assessing occupational radiation exposure [17,31–34], but little is known about orthopaedic surgeons. To our knowledge, this is the first study in Japan to use cytogenetic markers to evaluate their exposure. Our findings revealed that most surgeons in our study had higher dicentric frequencies than the general population [18, 25], where typically one or two dicentrics are detected per 1000 cells [18]. Similarly, the frequency of observed translocations among in 16 out of 17 surgeons exceeded the expected levels for their respective ages [23]. These elevated yields of dicentric chromosomes and translocations strongly suggest some degree of exposure to ionizing radiation, particularly considering that the observed frequencies in our study were higher than those previously reported in other professions with occupational radiation exposure (Table 3). Interventional radiologists in Korea [32] and radiologists in the U.S [35] were the only professional groups with higher chromosomal damage compared to orthopaedic surgeons in our study.

**Table 3:** Comparison of dicentric frequency (DF), rings and acentric fragments (frags) per 1000 metaphase cells, and translocations (Tr) in workers occupationally exposed to ionizing radiation

Occupation	Number of subjects	Number of cells analysed	Chromosome aberration frequencies^a^			
			DF	Rings	Frags	Tr
Bigatti *et al.* [36] (no physical dosimetry)						
General public	30	2920	0.3	0	–^b^	–
Radio diagnostics workers	63	6254	1.6	0.1	–	–
Tawn e*t al.* [37]						
Nuclear facility workers (exposed to <50 mSv)	39	3900	1.0	0	2.1	0.82
Nuclear facility workers (exposed to 140–560 mSv)	17	1700	1.2	0	2.9	0.88
Nuclear facility workers (exposed to >760 mSv)	14	1400	1.86	0	2.1	1.86
Kašuba *et al.* [31] (no physical dosimetry)						
General public	200	40 000	0.5	0.05	2.1	–
Anesthesiologists	80	16 000	1.44	0.25	9.06	–
Anesthetic technicians	45	9000	0.89	0	6.78	–
Radiology technicians	250	50 000	0.74	0.08	5.5	–
Surgeons	100	20 000	1.15	0.05	6.15	–
Nurses	50	10 000	1.1	0	5.7	–
Radiologists	100	20 000	0.6	0.05	6	–
Urologists and gynecologists	40	8000	2.13	0.13	6.13	–
Montoro e*t al.* [35]						
Radiologists (exposed to 2.2–228 mSv)	9	17 626	5.6	–	–	2.67^c^
Sigurdson e*t al.* [38]						
Radiologic technologists (exposed to <10 mGy^d^)	53	95400^e^	–	–	–	1.1
Radiologic technologists (exposed to >10–20 mGy)	43	77 400	–	–	–	1.3
Radiologic technologists (exposed to >20–30 mGy)	27	48 600	–	–	–	1.7
Radiologic technologists (exposed to >30–40 mGy)	17	30 600	–	–	–	1.5
Radiologic technologists (exposed to >40 mGy)	10	18 000	–	–	–	1.7
Tawn *et al.* [39]						
General public	66	6600	0.61	0.15	3.24	–
Tritium workers (0.25–87 mGy)	34	6800	1.91	0.15	2.12	–
Lee *et al.* [32]						
General public	77	77 000	1.34	–	–	–
Radiographers (0–79.2 mGy)	120	12 000	2.42	–	–	–
Interventional radiologists (0.1–96.8 mGy)	52	52 000	4.98	–	–	–
This study						
Orthopaedic surgeons	18	32 573	3.2	0.58	9.06	1.9
Abe et. al [25]						
Japanese population	5	10 000	1.0	–	–	0.5

^a^Aberrations per 1000 cells or 1000 CE for Tr

^b^Dashed line indicates the result was not reported in study

^c^Montoro *et al*. [35] evaluated 9722 metaphase cells total (as if the full genome)

^d^Sigurdson e*t al.* [38] estimated occupational radiation dose to the red bone marrow

^e^Sigurdson e*t al.* [38] evaluated 1800 metaphase cells per subject (as if the full genome had been scored)

In Japan, the age-adjusted incidence rate of SCC is ~3.40 per 100 000 persons (0.0034%) [36], while melanonychia, characterized by brown or black nail discoloration, affects ~10% to 20% of the population [37]. Among the orthopaedic surgeons in our study, 10% had SCC, 55% had melanonychia, and their dicentric frequencies were five to three times higher than those of the general population. Because malignant conditions such as squamous cell carcinoma or Bowen’s disease can themselves contribute to chromosomal instability, these findings should be interpreted with caution. Nevertheless, the increased frequencies of dicentrics and translocations observed in most surgeons, combined with 15 out of 18 experiencing adverse effects such as pigmentation changes, chronic radiation dermatitis and SCC on their fingers, would indicate that there is a need for improved radiation protection measures.

In our analysis, 111 dicentrics were observed in 32 573 cells while 345 total apparent simple translocations were observed in 45 674 cells (18 031 CE). In surgeons showing aberration yield above their respective decision threshold, the estimated doses were 75 ± 24 and 321 ± 104 mGy, respectively. In all surgeons except two, dose estimates from translocations were higher than dicentrics. This discrepancy is typical when estimated doses are compared between both biomarkers [35, 40] and could potentially be attributed to several factors. Dicentric chromosomes are unstable aberrations; affected lymphocytes are prone to apoptosis, leading to underestimation of dose if blood is collected too long after exposure [41]. In contrast, spontaneous translocations are more stable and may accumulate over time, though their frequency can also be influenced by confounding factors such as smoking [38], contributing to higher dose estimates via translocations. Medical exposures, including diagnostic imaging procedures like CT scans, may also contribute to both dicentric and translocation formation [24, 26, 42]. However, since dicentrics are short-lived, their detection is unlikely unless blood is drawn shortly after such procedures. Additionally, inter-individual variability in biological responses to ionizing radiation, shaped by genetic, environmental, lifestyle and medical factors, further complicates dose estimation [43].

Neither biomarker had correlation with work experience or age of the surgeon (Fig. 3, Supplementary Fig. S3). This was particularly unexpected for translocations because they tend to accumulate with time as stable aberrations can be passed down to daughter cells. In our study group, any ionizing radiation exposure received by the surgeons was most likely partial irradiation to the hands or fingers [44]. The adverse effects reported by the surgeons, including discoloration, would occur by an accumulation of doses to the hands. However, dicentric distributions from all surgeons followed a Poisson distribution [45] and either the doses were too low and/or too long has passed from exposure. Additionally, there are fewer hematopoietic stem cells (HSCs) in the hand compared to the bone marrow of the flat and long bones, and thus the translocations are likely limited to the life span of circulating lymphocytes. The HSCs, responsible for continuous production of all types of blood cells throughout an individual’s lifetime, are mostly unirradiated and do not contribute to the continuous production of circulating lymphocytes with translocations. This type of partial body irradiation could cause a shorter lifespan of translocations and elucidate why there was no trend between translocation frequency and surgical experience or age. It is also plausible that with increased experience, surgeons may have encountered fewer partial irradiation scenarios to the hands due to more training. A study of interventional radiologists in South Korea found a significant association between the total duration of all interventional procedures per week and the estimated doses based on the dicentric yield [32]. In this study, we did not have information on the total duration of procedures performed for each subject and a more comprehensive future study should examine both years of practice and detailed protective habits to better understand their relationship to chromosomal aberration frequency, and to evaluate the effectiveness of current radiation safety measures.

Three orthopedic surgeons in this study showed no apparent radiation-related health effects despite long clinical experiences. These individuals also exhibited relatively lower frequencies of chromosome aberrations compared to others with similar exposure histories (Table 2). This pattern may reflect individual differences in radiosensitivity or variations in adherence to radiation protection practices. However, the small number of unaffected cases limits the ability to draw firm conclusions, highlighting the need for larger studies that include more surgeons without adverse effects.

The study has several limitations. Most critically, individual dosimetry data were unavailable for participants (e.g. Hp(10), Hp(0.07) or ring/badge dosimeters), limiting the ability to directly correlate dose history with cytogenetic damage. More importantly, we cannot conclusively attribute the increase in chromosomal aberrations to radiation exposure; rather, our findings indicated a strong association that warrants further investigation. Self-reported adherence to dose limits, without verification from dosimeter records or information on dosimeter placement, introduces potential recall bias and underreporting of localized exposure. Furthermore, partial-body exposures to the hands complicated dose estimation, as the standard dicentric dose-response curve assumes whole-body uniform exposure within a specific post-irradiation timeframe. While translocations are more stable over time, they are influenced by confounding factors such as smoking. No correlation between chromosomal damage and years of surgical experience or age suggested the need for more precise monitoring of cumulative partial-body doses, particularly for the hands. However, information on the annual number of X-ray guided surgeries was not available in this study and should be considered in future investigations to better assess exposure-related risk. Additionally, observational or audit data to verify reported use of PPE was not collected and this limited our ability to objectively assess compliance and its relationship to chromosomal damage. The lack of correlation might also be due to behavioural confounders such as variation in PPE use (e.g. lead apron only vs. full protection including gloves and thyroid shields), differences in fluoroscopy procedural settings, and operator habits. In addition, the small sample size (*n* = 18) limited the generalizability of our findings.

Despite these limitations, the findings highlight the urgent need for enhanced radiation protection in fluoroscopic procedures. Japan currently lacks a radiation worker classification system equivalent to the Category A and B classifications defined by EURATOM [1]. Although orthopaedic surgeons are generally required to comply with institutional radiation safety protocols [2], there is considerable variation in the enforcement and implementation of these guidelines across hospitals and clinics. To address this, stricter protocols and better resources are needed. Effective strategies for mitigating exposure include mandatory use of ring dosimeters, optimizing procedural techniques to reduce beam-on time, and increasing the distance from radiation sources [46]. Additionally, advancements in robotics hold promise for minimizing direct hand exposure [47]. Routine biodosimetry assessments should also be integrated into occupational health monitoring to detect early biomarkers of radiation exposure. New regulations and radiation protection guidelines have been established and enforced in recent years, aiming to address these concerns. Future research should evaluate whether recent radiation protection measures have led to a measurable reduction in chromosomal aberrations and overall improvements in radiation safety practices in Japan. Importantly, future studies should include verified personal dosimetry data, such as badge and ring dosimeter readings, to determine whether cytogenetic biomarkers correlate with actual cumulative dose and to assess the effectiveness of current protective protocols in minimizing occupational exposure over time.

In conclusion, our study identified elevated chromosomal damage in orthopaedic surgeons performing fluoroscopic procedures, despite self-reported compliance with dose limits. The observed biomarkers and adverse health effects suggests increased radiation exposure, warranting increased awareness and protective measures. While the study provides valuable insights, future research should integrate comprehensive dosimetric data and evaluate long-term health outcomes, thereby enhancing radiation safety culture and ensuring the well-being of healthcare professionals.

## Supplementary Material

Supplemental_word_clean_rraf085
