# Supplementary material for: Occupational radiation exposure indicated by increased chromosomal damage in lymphocytes of orthopaedic surgeons in Japan
Source: J Radiat Res. 2026 Jan 13;67(1):68–77. doi: 10.1093/jrr/rraf085 (PMC12856030; doi:10.1093/jrr/rraf085)
Supplement: Supplemental_word_clean_rraf085 [file supplemental_word_clean_rraf085.docx]

Surgeon ID for study: _______________________ Date: __________________________

Age: __________________________

Gender: __________________________

Do you smoke (Y/N)?________________

If yes, how many cigarettes per day? ________________

How many years of orthopaedics training/experience do you have? ______ (Years)

Do you drink alcohol? (Y/N)? _____________
 If yes, how much per day (mL)? _________

Do you wear:

1. A lead apron (Y/N)?________________
2. Film badge personal dosimeters (Y/N)?________________
   1. Positioned on your chest (Y/N)?________________
   2. If you have a dosimeter, do you know the results? (Y/N) _________
3. Ring dosimeter (Y/N)?________________

To the best of your knowledge, did you exceed the recommended dose limit of 100 mSv per five years or 50 mSv in any single year? (Y/N) _________

Do you have any adverse effects or medical conditions (Y/N)?________________

If yes, what medical condition(s)? _________________________________

**Supplementary Fig. S1.** Questionnaire provided to surgeons in this study. The survey has been translated from Japanese to English.

**Supplementary Fig. S2.** Dicentric frequency per cell by smoking habit, i.e., Brinkman’s index. The 95% CI of all data is shown by the shaded grey area.


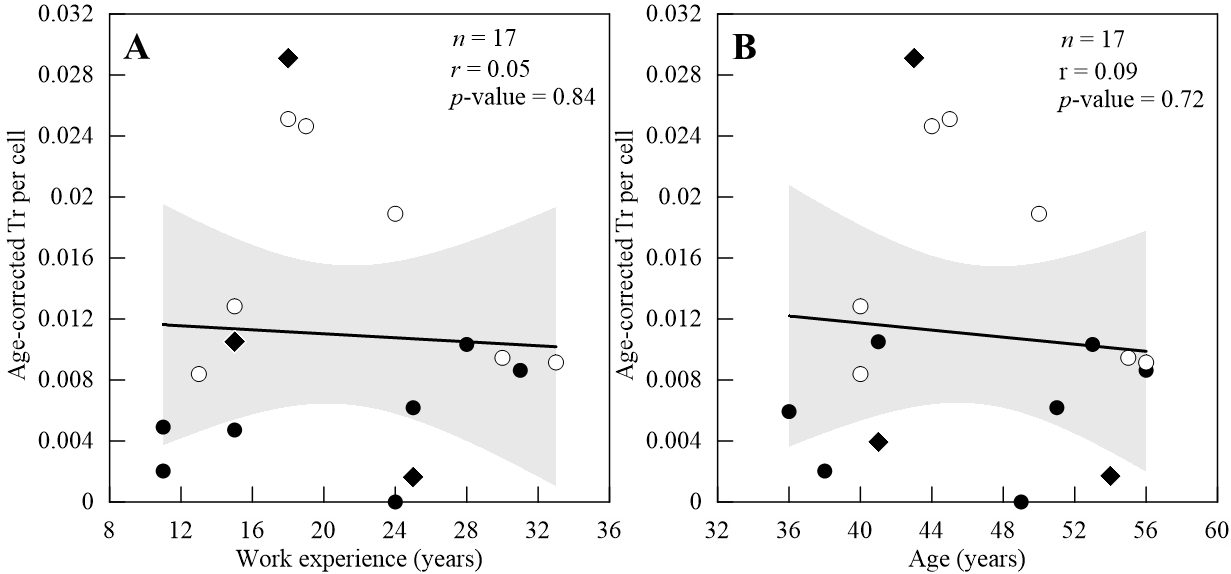


**Supplementary Fig. S3.** *Figure S2a* Age-corrected translocation frequency per cell equivalent by work experience (years) of male orthopaedic surgeons. *Figure S2b* Age-corrected translocation frequency per cell equivalent by age (years). In both graphs, closed black circles indicate non-smokers, open circles indicate smokers, and the black diamond had no smoking information. In both graphs, the 95% CI of all data is shown by the shaded grey area.

| ID | Sex | Age | Years of Experience | Smoking index | Giemsa analyzed slides | | | | | | |
| --- | --- | --- | --- | --- | --- | --- | --- | --- | --- | --- | --- |
|  |  |  |  |  | No. of analyzed metaphases | Types of aberrations | | | | Dic per cell | Estimated dose (mGy) Obtained with Dose Estimate v5.2 |
|  |  |  |  |  |  | # of cells with CA | No. of rings | No. of Frags | No. of dics |  |  |
| HKX001 | M | 53 | 28 | 0 | 2551 | 28 | 2 | 27 | 11 | 0.004 | 51 |
| HKX002 | M | 50 | 24 | 310 | 1815 | 17 | 1 | 8 | 4 | 0.002 | 12* |
| HKX003 | M | 44 | 19 | 220 | 1537 | 25 | 0 | 13 | 7 | 0.005 | 55* |
| HKX004 | M | 54 | 25 | N/A | 1389 | 27 | 1 | 7 | 1 | 0.001 | 0* |
| HKX005 | M | 40 | 15 | 225 | 2003 | 44 | 3 | 30 | 10 | 0.005 | 63 |
| HKX006 | M | 45 | 18 | 100 | 1801 | 25 | 0 | 13 | 5 | 0.003 | 23* |
| HKX007 | M | 44 | 19 | 540 | 2001 | 29 | 2 | 20 | 8 | 0.004 | 45* |
| HKX008 | M | 49 | 24 | 0 | 2000 | 28 | 1 | 8 | 3 | 0.002 | 0* |
| HKX009 | M | 56 | 33 | 200 | 1000 | 10 | 0 | 3 | 2 | 0.002 | 9* |
| HKX010 | M | 40 | 13 | 100 | 2000 | 31 | 1 | 20 | 6 | 0.003 | 27* |
| HKX011 | M | 38 | 11 | 0 | 1838 | 34 | 0 | 22 | 6 | 0.003 | 32* |
| HKX012 | M | 51 | 25 | 0 | 1691 | 23 | 0 | 9 | 3 | 0.002 | 5* |
| HKX013 | M | 36 | 11 | 0 | 1827 | 19 | 1 | 13 | 5 | 0.003 | 22* |
| HKX014 | M | 41 | 15 | N/A | 1114 | 16 | 0 | 5 | 1 | 0.001 | 0* |
| HKX015 | M | 56 | 31 | 0 | 2000 | 30 | 3 | 32 | 7 | 0.004 | 36* |
| HKX016 | M | 55 | 30 | 152 | 2000 | 27 | 1 | 22 | 12 | 0.006 | 81 |
| HKX017 | M | 43 | 18 | N/A | 2006 | 36 | 2 | 33 | 15 | 0.007 | 106 |
| HKX018 | M | 41 | 15 | 0 | 2000 | 29 | 1 | 20 | 5 | 0.003 | 18* |

**Supplementary Table. S1.** Information from surgeons and Giemsa analysed slides. * indicates estimated doses are inaccurate as dicentrics are not above the decision threshold

| ID | FISH analyzed slides | | | | | | | | | | |  |  |
| --- | --- | --- | --- | --- | --- | --- | --- | --- | --- | --- | --- | --- | --- |
|  | No of metaphases | Whole genome equivalent (CE) | No. of Tr | Bg Tr/CE | Tr/CE | Age-corrected Tr/CE | Estimated dose (mGy) Obtained with curve eqn | | Decision threshold (Tr) Obtained with ISO 20046 | |  |  |  |
|  |  |  |  |  |  |  |  |  |  |  |  | |  |
| HKX001 | 131 | 51.7178 | 1 | 0.009005951 | 0.019 | 0.01032977 | | 246* | | 2 | |  | |
| HKX002 | 3171 | 1251.89 | 34 | 0.008260923 | 0.027 | 0.018898112 | | 364 | | 17 | |  | |
| HKX003 | N/A | N/A | - | - | - | - | | - | | - | |  | |
| HKX004 | 3001 | 1184.77 | 13 | 0.009263086 | 0.011 | 0.001709501 | | 139* | | 18 | |  | |
| HKX005 | 3221 | 1271.63 | 24 | 0.006044337 | 0.019 | 0.012829151 | | 284 | | 14 | |  | |
| HKX006 | 1966 | 776.161 | 25 | 0.007103328 | 0.032 | 0.025106481 | | 433 | | 11 | |  | |
| HKX007 | 3857 | 1522.71 | 48 | 0.006883876 | 0.032 | 0.024638813 | | 428 | | 17 | |  | |
| HKX008 | 3324 | 1312.29 | 10 | 0.008021158 | 0.008 | 0 | | 0* | | 17 | |  | |
| HKX009 | 1605 | 633.641 | 12 | 0.009790938 | 0.019 | 0.009147226 | | 226* | | 12 | |  | |
| HKX010 | 3335 | 1316.63 | 19 | 0.006044337 | 0.014 | 0.00838643 | | 213 | | 14 | |  | |
| HKX011 | 1650 | 651.407 | 5 | 0.005646742 | 0.008 | 0.002028953 | | 65* | | 8 | |  | |
| HKX012 | 2759 | 1089.23 | 16 | 0.008504934 | 0.015 | 0.006184327 | | 171* | | 16 | |  | |
| HKX013 | 679 | 268.064 | 3 | 0.005263349 | 0.011 | 0.005928018 | | 51* | | 4 | |  | |
| HKX014 | 3983 | 1572.46 | 16 | 0.006248595 | 0.010 | 0.003926567 | | 144* | | 16 | |  | |
| HKX015 | 2337 | 922.629 | 17 | 0.009790938 | 0.018 | 0.008634672 | | 217 | | 16 | |  | |
| HKX016 | 3203 | 1264.52 | 24 | 0.009524726 | 0.019 | 0.009454826 | | 231 | | 19 | |  | |
| HKX017 | 3824 | 1509.68 | 54 | 0.006668308 | 0.036 | 0.029100753 | | 473 | | 17 | |  | |
| HKX018 | 3628 | 1432.31 | 24 | 0.006248595 | 0.017 | 0.010507609 | | 249 | | 15 | |  | |

**Supplementary Table. S2.** Information from surgeons and translocation analysed slides. * indicates estimated doses are inaccurate as observed Tr are not above the decision threshold.

| Steps | Predictors | β | B | SE(B) | *t* | *p*-value | | 95% CI | | R | Adjusted R |
| --- | --- | --- | --- | --- | --- | --- | --- | --- | --- | --- | --- |
|  |  |  |  |  |  |  |  | upper | lower |  |  |
| Step1 | Constants | – | -1.716E-19 | 3.593E-04 | 0 | 1 |  | -8.006E-04 | 8.006E-04 | 0.041 | -0.246 |
|  | Age | -4.922E-01 | -8.456E-05 | 4.459E-04 | -0.190 | 0.853 |  | -1.078E-03 | 9.090E-04 |  |  |
|  | Work Experience | 5.394E-01 | 8.517E-05 | 4.103E-04 | 0.208 | 0.840 |  | -8.289E-04 | 9.993E-04 |  |  |
|  | Smoking | 1.710E-01 | 1.288E-06 | 2.406E-06 | 0.535 | 0.604 |  | -4.074E-06 | 6.650E-06 |  |  |
| Step2 | Constants | – | -8.995E-04 | 7.262E-04 | -1.239 | 0.255 |  | -2.617E-03 | 8.178E-04 | 0.362 | -0.186 |
|  | Age | 1.259E+00 | 2.163E-04 | 5.203E-04 | 0.416 | 0.690 |  | -1.014E-03 | 1.447E-03 |  |  |
|  | Work Experience | -1.468E+00 | -2.317E-04 | 4.824E-04 | -0.480 | 0.646 |  | -1.373E-03 | 9.091E-04 |  |  |
|  | Smoking | 3.847E-01 | 2.898E-06 | 3.019E-06 | 0.960 | 0.369 |  | -4.241E-06 | 1.004E-05 |  |  |
|  | Age:Working Experience | 6.631E-01 | 2.049E-05 | 1.350E-05 | 1.518 | 0.173 |  | -1.143E-05 | 5.240E-05 |  |  |
|  | Age:Smoking | 1.027E+00 | 1.577E-06 | 4.238E-06 | 0.372 | 0.721 |  | -8.444E-06 | 1.160E-05 |  |  |
|  | Working Experience:Smoking | -1.643E+00 | -2.488E-06 | 4.213E-06 | -0.590 | 0.573 |  | -1.245E-05 | 7.475E-06 |  |  |
| Step3 | Constants | – | -9.886E-04 | 7.810E-04 | -1.266 | 0.252 |  | -2.900E-03 | 9.224E-04 | 0.393 | -0.314 |
|  | Age | 1.779E+00 | 3.056E-04 | 5.706E-04 | 0.536 | 0.611 |  | -1.091E-03 | 1.702E-03 |  |  |
|  | Work Experience | -2.141E+00 | -3.380E-04 | 5.422E-04 | -0.623 | 0.556 |  | -1.665E-03 | 9.886E-04 |  |  |
|  | Smoking | 2.794E-01 | 2.104E-06 | 3.479E-06 | 0.605 | 0.567 |  | -6.409E-06 | 1.062E-05 |  |  |
|  | Age:Working Experience | 8.208E-01 | 2.536E-05 | 1.665E-05 | 1.523 | 0.179 |  | -1.540E-05 | 6.611E-05 |  |  |
|  | Age:Smoking | 1.622E+00 | 2.491E-06 | 4.751E-06 | 0.524 | 0.619 |  | -9.133E-06 | 1.412E-05 |  |  |
|  | Working Experience:Smoking | -2.339E+00 | -3.541E-06 | 4.818E-06 | -0.735 | 0.490 |  | -1.533E-05 | 8.247E-06 |  |  |
|  | Age:Working Experience:Smoking | 3.084E-01 | 5.985E-08 | 1.067E-07 | 0.561 | 0.595 |  | -2.013E-07 | 3.210E-07 |  |  |

**Supplementary Table S3.** Hierarchical multiple regression analyses for testing interaction for Dic frequency on age, working experience, and smoking history

| Steps | Predictors | β | B | SE(B) | *t* | *p*-value | | 95% CI | | R | Adjusted R |
| --- | --- | --- | --- | --- | --- | --- | --- | --- | --- | --- | --- |
|  |  |  |  |  |  |  |  | upper | lower |  |  |
| Step1 | Constants | – | -2.211E-18 | 1.393E-03 | 0.000 | 1.000 |  | -3.104E-03 | 3.104E-03 | 0.637 | 0.529 |
|  | Age | 2.747E+00 | 2.975E-03 | 1.729E-03 | 1.721 | 0.116 |  | -8.762E-04 | 6.827E-03 |  |  |
|  | Work Experience | -2.576E+00 | -2.564E-03 | 1.590E-03 | -1.612 | 0.138 |  | -6.108E-03 | 9.795E-04 |  |  |
|  | Smoking | 7.851E-01 | 3.728E-05 | 9.328E-06 | 3.997 | 0.003 | ** | 1.650E-05 | 5.807E-05 |  |  |
| Step2 | Constants | – | 1.195E-03 | 3.370E-03 | 0.355 | 0.733 |  | -6.774E-03 | 9.164E-03 | 0.654 | 0.358 |
|  | Age | 2.158E+00 | 2.337E-03 | 2.415E-03 | 0.968 | 0.365 |  | -3.372E-03 | 8.047E-03 |  |  |
|  | Work Experience | -2.001E+00 | -1.992E-03 | 2.239E-03 | -0.890 | 0.403 |  | -7.286E-03 | 3.302E-03 |  |  |
|  | Smoking | 7.596E-01 | 3.607E-05 | 1.401E-05 | 2.575 | 0.037 | * | 2.942E-06 | 6.920E-05 |  |  |
|  | Age:Working Experience | -1.032E-01 | -2.010E-05 | 6.263E-05 | -0.321 | 0.758 |  | -1.682E-04 | 1.280E-04 |  |  |
|  | Age:Smoking | 5.454E-01 | 5.281E-06 | 1.966E-05 | 0.269 | 0.796 |  | -4.122E-05 | 5.178E-05 |  |  |
|  | Working Experience:Smoking | -5.802E-01 | -5.537E-06 | 1.955E-05 | -0.283 | 0.785 |  | -5.177E-05 | 4.069E-05 |  |  |
| Step3 | Constants | – | 1.313E-03 | 3.710E-03 | 0.354 | 0.736 |  | -7.766E-03 | 1.039E-02 | 0.656 | 0.254 |
|  | Age | 2.049E+00 | 2.219E-03 | 2.711E-03 | 0.819 | 0.444 |  | -4.414E-03 | 8.852E-03 |  |  |
|  | Work Experience | -1.860E+00 | -1.852E-03 | 2.576E-03 | -0.719 | 0.499 |  | -8.154E-03 | 4.451E-03 |  |  |
|  | Smoking | 7.817E-01 | 3.712E-05 | 1.653E-05 | 2.246 | 0.066 |  | -3.326E-06 | 7.756E-05 |  |  |
|  | Age:Working Experience | -1.363E-01 | -2.654E-05 | 7.912E-05 | -0.335 | 0.749 |  | -2.201E-04 | 1.671E-04 |  |  |
|  | Age:Smoking | 4.204E-01 | 4.070E-06 | 2.257E-05 | 0.180 | 0.863 |  | -5.115E-05 | 5.930E-05 |  |  |
|  | Working Experience:Smoking | -4.340E-01 | -4.142E-06 | 2.289E-05 | -0.181 | 0.862 |  | -6.015E-05 | 5.186E-05 |  |  |
|  | Age:Working Experience:Smoking | -6.477E-02 | -7.923E-08 | 5.07E-07 | -0.156 | 0.881 |  | -1.320E-06 | 1.162E-06 |  |  |

**Supplementary Table S4.** Hierarchical multiple regression analyses for testing interaction for Tr frequency on age, working experience, and smoking history.
